# The Gut Microbiome Alterations and Inflammation-Driven Pathogenesis of Alzheimer’s Disease—a Critical Review

**DOI:** 10.1007/s12035-018-1188-4

**Published:** 2018-06-23

**Authors:** Marta Sochocka, Katarzyna Donskow-Łysoniewska, Breno Satler Diniz, Donata Kurpas, Ewa Brzozowska, Jerzy Leszek

**Affiliations:** 1 Laboratory of Virology, Department of Immunology of Infectious Diseases, Hirszfeld Institute of Immunology and Experimental Therapy, Polish Academy of Sciences, Wroclaw, Poland; 2 Laboratory of Parasitology, General Karol Kaczkowski Military Institute of Hygiene and Epidemiology, Warsaw, Poland; 3 Department of Psychiatry and Behavioral Sciences, and The Consortium on Aging, The University of Texas Health Science Center at Houston, Houston, TX USA; 4 Department of Family Medicine, Wroclaw Medical University, Wroclaw, Poland; 5 Laboratory of Medical Microbiology, Department of Immunology of Infectious Diseases, Hirszfeld Institute of Immunology and Experimental Therapy, Polish Academy of Sciences, Wroclaw, Poland; 6 Department of Psychiatry, Wroclaw Medical University, Wroclaw, Poland

**Keywords:** Alzheimer’s disease, Gut microbiome, Neuroinflammation, Microbial amyloid, Therapeutic intervention

## Abstract

One of the most important scientific discoveries of recent years was the disclosure that the intestinal microflora takes part in bidirectional communication between the gut and the brain. Scientists suggest that human gut microflora may even act as the “second brain” and be responsible for neurodegenerative disorders like Alzheimer’s disease (AD). Although human-associated microbial communities are generally stable, they can be altered by common human actions and experiences. Enteric bacteria, commensal, and pathogenic microorganisms, may have a major impact on immune system, brain development, and behavior, as they are able to produce several neurotransmitters and neuromodulators like serotonin, kynurenine, catecholamine, etc., as well as amyloids. However, brain destructive mechanisms, that can lead to dementia and AD, start with the intestinal microbiome dysbiosis, development of local and systemic inflammation, and dysregulation of the gut-brain axis. Increased permeability of the gut epithelial barrier results in invasion of different bacteria, viruses, and their neuroactive products that support neuroinflammatory reactions in the brain. It seems that, inflammatory-infectious hypothesis of AD, with the great role of the gut microbiome, starts to gently push into the shadow the amyloid cascade hypothesis that has dominated for decades. It is strongly postulated that AD may begin in the gut, and is closely related to the imbalance of gut microbiota. This is promising area for therapeutic intervention. Modulation of gut microbiota through personalized diet or beneficial microbiota intervention, alter microbial partners and their products including amyloid protein, will probably become a new treatment for AD.

## Inflammation-Driven Ad Pathogenesis

Alzheimer’s disease (AD) is the most common form of dementia in older adults. It is a major public health problem, and the incidence and prevalence are expected to achieve epidemic proportions in the next few decades if no intervention aimed to prevent or reduce the disease progress is developed.

In early 90’s, the amyloid cascade hypothesis has been the main hypothesis about the AD pathophysiology AD [1]. Currently, it is widely accepted that the amyloid pathology may begin 10–20 years before the first symptoms of cognitive decline and the diagnosis of the AD. However, the amyloid deposition in the brain is not the sole culprit for the development of dementia. One of the consequences of amyloid deposition in the brain is the activation of immune responses in the brain. Amyloid-β (Aβ) deposits activate the microglia, the brain innate immune cells, leading to an inflammatory response in the central nervous system - CNS (neuroinflammation). An acute, self-limiting neuroinflammatory response leads to Aβ clearance, what is beneficial to neuronal protection [2, 3]. However, during aging process, brain certain changes occur in immune system (immunosenescence) and microglia-related immune response [4]. Persistent microglia activation (reactive microglia) leads to chronic inflammatory response that supports the neurotoxic processes, which cause brain injury and neuronal death [5, 6]. Moreover, reactive microglia activates astrocytes, another important glial cell that supports neuron functioning. Reactive astrocytes, as well as reactive microglia, contribute to neuroinflammatory burden and blood-brain barrier (BBB) dysfunction. BBB, an important CNS protection system, is responsible for the strict control of the molecules transported in and out of the brain. As we age, this perfectly working machinery weakens, facilitating the crossing of a large spectrum of pathogens (viruses, bacteria, fungi), immune cells, and their products into the brain [5, 7]. Inflammation-mediated BBB decay and unsettled cerebral endothelial layer maturation might be dependent on alterations in the gut microbiome. Thus, this supports the hypothesis about an association of chronic infections with neurodegenerative or neurodevelopmental diseases like AD [8].

**Fig. 1:**
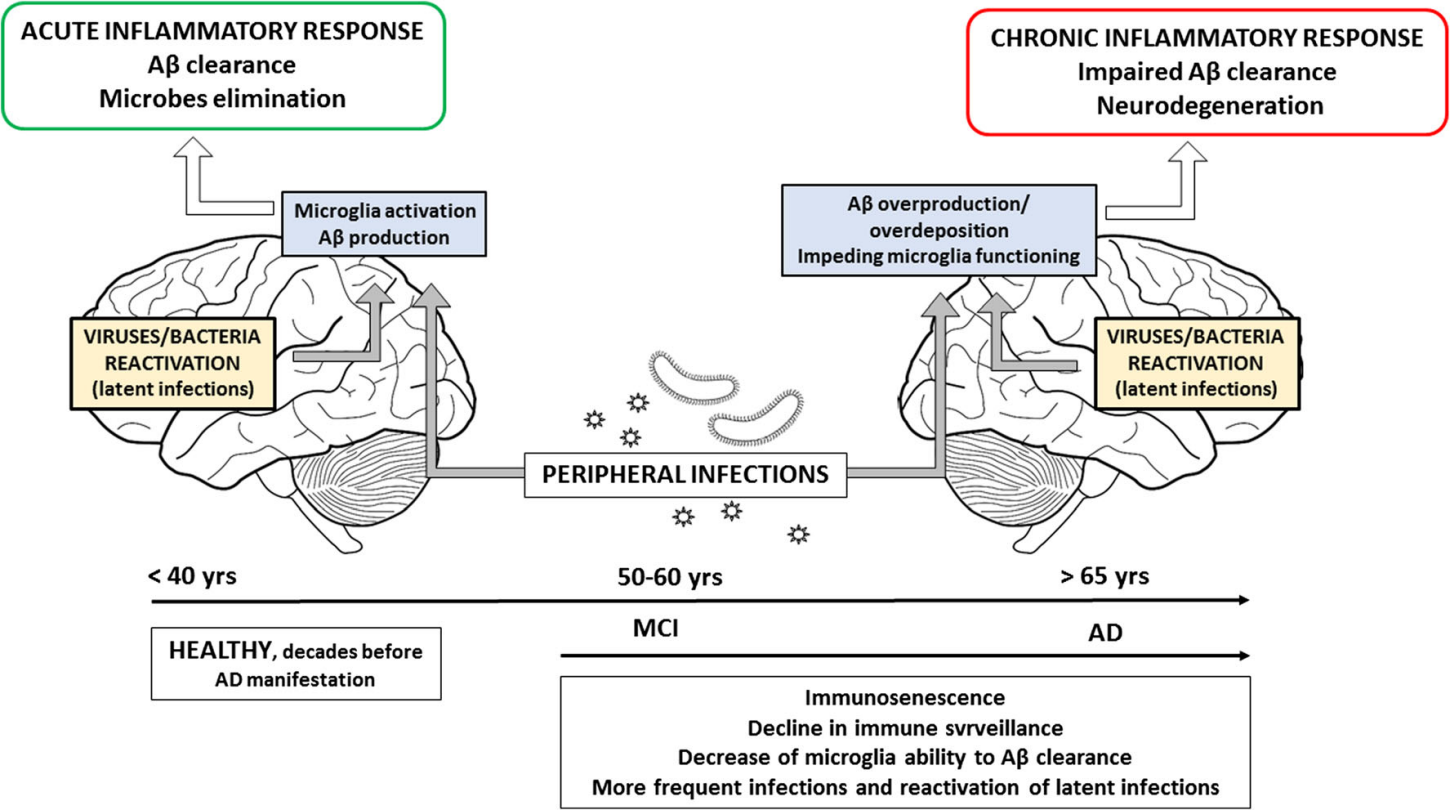
Age-related changes in the neuroinflmmatory response in the CNS—the role of local and peripheral infections. In healthy brain proper functioning of the brain innate immune response results in Aβ clearance and pathogen elimination [primary infection/reactivation]. Aging leads to the decline in immune surveillance and decrease of microglia fagocytic activity. Aβ overproduction/overdeposition with lose/limited antimicrobial capacity, as well as varied microbes and their products [LPS, amyloids] infecting or infiltrating into the brain from periphery, initiates the cascade of chronic neuroinflammatory reactions and neurodegenerative changes that can cause AD

There are many doubts and controversies about amyloid cascade hypothesis, and it is more and more likely it may fail due to rising documented studies showing no correlation between Aβ deposits and clinical manifestation of the disease [9, 10]. Many patients with dementia have no Aβ deposits in the brain, on the other hand, the brains of elderly non-demented patients reveal as much senile plaques as that of dementia patients [11–13]. There are strong suggestions that Aβ amyloid deposition is an aging-related phenomenon, fully independent, and unrelated with the onset of AD, which is in contrary to the amyloid hypothesis [10]. Recently, the inflammatory hypothesis of AD becomes more and more significant [14]. AD is considered a systemic disease because it is related to neuroinflammation in the brain as well as to inflammatory reactions in the periphery, and it is suggested. Although, the most important is that the inflammatory processes in the brain could be initiated and act many years before senile plaques appear [7, 14], and that Aβ production is connected with an antimicrobial response. An antimicrobial activity of Aβ (act as an antimicrobial peptide – AMP) against fungi, bacteria, and viruses, like HHV-1 was demonstrated [15, 16]. Thus, these studies shed a light on the infectious hypothesis of AD and the role of infectious agents in neuroinflammation. We can imagine that Aβ is produced in every new infection (microbes passage from periphery into the brain) or reactivation of microbes from latent infection in brain tissue, which affects the brain periodically. Additionally, aging is the reason of immunosenescence, overall immune response decline, and decrease of microglia ability to correct Aβ clearance or pathogens phagocytosis. Thus, during years, these conditions lead to inflammatory response switch from beneficial to detrimental (chronic inflammation) [5]. Taking above into consideration, it is more clear that Aβ overproduction causes overdeposition as senile plaques with lose/limited antimicrobial capacity of Aβ. It seems that Aβ deposits as well as different microbes and their products [e.g., LPS, amyloids] infiltrating into the brain might be an initiating factor of neuroinflammation and neurodegenerative changes observed in AD that Aβ deposition (Aβ overproduction) is the initiating factor of neuroinflammation (Fig. 1).

More recently, there is an increasing interest to evaluate the role of the gut microbiome in the AD pathogenesis and the induction of neuroinflammatory changes. A recent advance in studies of AD etiology suggests that dysbiosis of microbiota (gut, mouth, nose) during life may lead to systemic inflammatory response and influence the immune response of microglia. Moreover, with time, the gut and the BBB permeability increases, and the vicious circle of neuronal destruction begins to work.

## Gut Microbiome

Lederberg and McCray for the first time in 2001 provided a new specific definition of the term “microbiome,” and its current usage describes the ecological community of commensal, symbiotic, and pathogenic microorganisms sharing human body space [17]. The term refers both, the microorganisms and their sets of activity. Each person harbors from 10 to 100 trillion symbiotic microbial cells, thousands of species, mainly localized in the human gut [18]. To the human microbiome, the microbiota (which includes bacteria, archaea, eukaryotic, viruses, bacteriophages, and eukaryotic microbes), their genomes and a full complement of microbial genes (at least 20 million unique microbial genes), and gene products are enlisted [19]. In 2007, the human microbiome project was established with the goal of understanding the roles that the symbiotic microorganisms play and how they affect human health [20]. We still know little about the biology of the microbiome and how to prime immunity and to maintain host health [21]. The body sites colonized by microbiome include the oral cavity, skin, and gastrointestinal tract as well as the vagina in females. As a part of the Human Microbiome Project, the site-specific microbial consortia genus have been studied [22].

The human gastrointestinal (GI) tract harbors a most abundant population of microorganisms. The reason is that the GI represents one of the largest interfaces (250–400 m^2^) between the host, environmental factors, and antigens in the human body [23]. Some study suggests that more than 5000 bacteria taxa may reside in the gut. They belong mostly to the bacterial phyla Firmicutes and Bacteroidetes [23]. The colonization of the gut in infants starts shortly after birth and depends on the delivery mode. Lactobacillus and Prevotella dominate in babies’ guts delivered via vaginal mode and contain similar microbiota as mother’s vagina. Babies delivered via Caesarian section (C-section) acquire microbiota similar to those typically associated with the skin (i.e., Propionibacterium, Staphylococcus, and Corynebacterium) [22]. It has been estimated the fecal microbiota of 72% of vaginally delivered infants resembles that of their mothers’ fecal microbiota; in babies delivered by C-section, this percentage is reduced to only 41% [23]. During the first year of life, the gut microbiota develops depending on diet (formula-fed or breastfed) and its diversity increases. By around 2.5 years of age, the composition, diversity, and functional capabilities of the infant microbiota resemble those of adult microbiota [22]. During the adulthood, the gut microflora seems to be relatively stable, till 65 years of age when the microbial community shifts, with an increased abundance of Bacteroidetes phyla and Clostridium cluster IV (in younger people cluster XIVa is more prevalent) [23].

### Dysbiosis

Dysbiosis of the microbiota and viruses cause some diseases from infections through liver diseases, gastrointestinal cancers, metabolic diseases, respiratory diseases, mental or psychological diseases, and autoimmune diseases [24]. David et al. [25] link over 10,000 longitudinal measurements of human wellness and action to the daily gut and salivary microbiota dynamics of two individuals over the course of 1 year. These time series show overall microbial communities to be stable for months. However, rare events in each subjects’ life rapidly and broadly impacted microbiota dynamics. Travel from the developed to the developing world in one subject led to a nearly twofold increase in the Bacteroidetes to Firmicutes ratio, which reversed upon return. Enteric infection in the other subject resulted in the permanent decline of most gut bacterial taxa, which were replaced by genetically similar species. Still, even during periods of overall community stability, the dynamics of select microbial taxa could be associated with specific host behaviors. Most prominently, changes in host fiber intake positively correlated with next-day abundance changes among 15% of gut microbiota members. The findings of David et al. [25] suggest that although human-associated microbial communities are stable, they can be quickly and profoundly altered by common human actions and experiences. The temporal dynamics of host-associated microbial communities (the microbiota) are of growing interest due to these communities’ relevance for health [26–30]. Normally, human microbiota remains stable for months, and possibly even years [31, 32]. However, studies across mice and humans suggest that common aspects of the modern Western lifestyle, including antibiotics [26, 33–35] and high-fat diets [27], can alter commensal microbial communities. In turn, those microbial disturbances may increase pathogen susceptibility [29], obesity [28, 36], and auto-inflammatory disease [30], diseases which are becoming more frequent in the developed world. Despite their potential health impact, a full list of lifestyle factors capable of altering human microbiota remains incomplete. Intervention studies are regularly performed to identify host behaviors that affect microbial dynamics, and they have notably demonstrated human gut microbial sensitivity to antibiotics [33–35], bowel surgery [37], and short-term diet shifts [38, 39]. However, interventions by design only test a small number of hypotheses; thus, a large, and potentially unfeasible, number of interventional studies are needed to fully explore the rich diversity of human actions and behaviors. An alternative approach for efficiently linking numerous host factors to microbial responses is to longitudinally observe both the host and microbiota and to infer relationships between them. Such observational studies have recently been used to show that menstrual cycles are the primary driver of vaginal microbial dynamics in women [40], and to show that infant gut microbiota begins transitioning towards adult communities after weaning [41]. In these time series, the quantity of host lifestyle variables that can be related to microbial dynamics is only bound by the number of host factors that can be tracked. Still, host tracking is non-trivial for ethical and logistical reasons, such as the need to repeatedly survey participants and the enforcement of subject compliance. Hence, many microbiome time series have featured limited longitudinal host metadata [32, 42], making it difficult to link microbial dynamics to host behavior.

Gut microbiota alterations can result from exposure to mentioned above lifestyle-related or environmental factors, but also to pathogens. The greatest potential to cause microbial dysbiosis has an enteric pathogens. However, gut microbiome is related to oral cavity flora, both in diversity and in composition. It is noticed that the swallowed saliva of patients with periodontitis may contain great amount of bacteria (up to 10^12^ bacteria/day) [43]. Thus, recent evidences are pointing on a causative link between oral pathogens (periopathogens) and changes in the intestinal microbiota composition as well as inflammatory changes in various tissues and organs including brain tissue [44]. In healthy oral cavity, commensals such as Actinomycetes and Streptococci, remain in physiological balance and do not cause the disease (symbiotic microbial community). Any disturbance of this ecological state may lead to the development of the disease such as periodontal disease with the presence of dysbiotic microbial community—composed with Firmicutes, Proteobacteria, Spirochaetes, and Bacteroidetes [45]. Periodontitis is linked with several systemic diseases and conditions like cardiovascular diseases (atherosclerosis), obesity, respiratory infections, adverse pregnancy outcome, rheumatoid arthritis, and diabetes mellitus [45, 46] as well as gut microbiota dysbiosis. Moreover, hyperactivated systemic inflammatory response, during periodontal disease, may in turn contribute with neuroinflammation and AD [47]. Thus, periodontitis is considered as a real additional source of oral bacterial pathogens and bacterial molecules like LPS, flagellin, peptidiglican or DNA, and pro-inflammatory molecules, and readily modifiable risk factor for AD [48, 49]. Periodontal inflammation can expand causing changes in the intestinal microflora and subsequently exacerbate host’s systemic inflammatory response. The proportions of phylum Bacteroidetes was significantly lower in fece after *P. gingivalis*-administration in mice, and the proportion of phylum Firmicutes was significantly higher. It seems that changes in the gut microbiota composition switched the gut immune profile to Th17 dominance [50]. Swallowing periodontal bacteria may influence on gut microbiota composition and induce inflammatory changes in various tissues and organs as well as translocation of gut microbiota into the liver. Oral administration of *P. gingivalis* in mouse model induced change of bacterial composition in the ileal microflora [43, 51], endotoxemia, and resulted in glucose-tolerance and insulin sensitivity decreased as well as increased in macrophage infiltration into the adipose tissue with much higher accumulation of fat and triglycerides. In the same investigation, authors [43] noted that infection with *P. gingivalis* caused inflammatory response in the liver through the increase expression of pro-inflammatory cytokines TNF-α, IL-6, as well as Fitm2 and Plin2 -associated with lipid droplet formation. The last important may support the thesis that periodontitis affects NAFLD (nonalcoholic fatty liver disease). Komazaki et al. [52] showed that anti-*Aggregatibacter actinomycetemcomitans* antibody titers correlated positively with visceral fat in NAFLD patients. Mouse treated with *A. actinomycetemcomitans* developed impaired glucose tolerance and insulin resistance. Studies showed that periodontitis may induce systemic low-grade inflammation. Experimental periodontitis in obese rats resulted with an increased gene expression of TNF-alpha and CRP in liver and increased levels of IL-6 and CRP in adipose tissue [53]. Thus, it is getting clear that chronic inflammation with low intensity of stimulating factor (chronic infections) may lead to the induction/aggravation of systemic inflammation, which subsequently increase neuroinflammation and cause neurodegenerative changes and AD. Recent advances in studies of oral health status as a potential contributor to inflammatory diseases have been reported here [54].

## The Gut-Brain Axis and Alzheimer’s Disease

Intestinal microflora, which contains up to 95% of all human microbiome bacteria, forms the microbiome-gut axis that provides two-way communication through cytokine, immunological, hormonal, and neuronal signals [55–57]. Since the intestinal mucosal lymphoid tissue contains 70–80% of the immune system in the whole body, it is considered to be the largest and most important human immune organ, and this large mass of mucosa-associated lymphoid tissue stays in continuous close contact with the enormous number of bacteria (even 100 trillion) of the human intestinal microbiome. It is well established that bacteria produce and secrete a range of compounds that reduce the tightness of the intestinal barrier, facilitating the contact of the intestinal microbiome with submucosal lymphoid tissue [58]. As a result, systemic inflammatory reactions occur, which impair the BBB and promote neuroinflammation and, ultimately, neurodegeneration [58–60]. Commensal microbiota produces a range of neuroactive molecules, such as serotonin, kynurenine, melatonin, GABA (gamma-aminobutyric acid), catecholamines, histamine, and acetylcholine [61, 62].

In particular, the role of the intestinal microbiome in the metabolism of tryptophan is relatively well documented, and some studies showed that dysregulation of serotonin and kynurenic pathways was documented in various neurodegenerative disorders, also in AD [63]. Dysregulation of kynurenine route of tryptophan pathway was suggested as a major contributor of AD [64, 65]. An important issue is the involvement of the peripheral and central/cerebral pool of tryptophan metabolites, which is associated with their penetration through the BBB. On the example of kynurenine, it was shown that various metabolites were transported differently across the BBB: both active transport, involving large neutral amino acid carrier (L-kynurenine, 3 hydroxykynurenine) as well as passive diffusion (anthranilic acid, kynurenic acid, and quinolinic acid) played a role in the transport of kynurenines to the brain [66]. On the other hand, it should be taken into account that dysregulation of the intestinal microflora leads to increased permeability of intestinal barriers and the BBB, resulting in increased penetration of products derived from microbial gut from the blood into the brain [58]. However, there is no doubt that the effect of the microbiome on the brain function is not a simple sum of the spectrum of all metabolites produced by bacteria because the different abilities of these metabolites to penetrate the BBB play a key role. A large number of different metabolites produced by gut microbiota may directly or indirectly affect brain function. Among them short-chain fatty acids (SCFAs), including acetate, butyrate, and propionate can modulate peripheral and central effect; *butyrate* affects cholinergic neurons of the gut nervous system, causing increased motility, whereas *propionate* seems to decrease motility and increases secretions. *Acetate* can cross the blood-brain barrier and signals satiety to the brain. Additionally, it affects microglia and also decreases permeability in the BBB [67–69]. Butyrate is a multi-functional molecule that exerts beneficial neuroprotective effects and improves brain health [70]. In addition to being an important substrate for energy generation, it increases mitochondrial respiration rate and ATP production and also inhibits/inhibits histone deacetylases, affecting both the functions of many genes and the functions of numerous cellular proteins. Also, many of its cellular effects are associated with specific binding to G protein-coupled receptors (FFAR3 and FFAR2; free fatty acid receptors 3 and 2) [70]. Butyrate is a potent agonist of FFAR3, whereas acetate and propionate are agonists of FFAR2. It should also be noted that the ketone body beta-hydroxybutyrate, although structurally similar to butyrate, unlike butyrate inhibits the functions of the FFAR3 receptor and decreases the activity of neurons [70]. It was also described that in mice, models of traumatic brain injury, intra-peritoneal injection of sodium butyrate attenuated neuronal deficits and brain edema and restored the blood-brain barrier [71]. In mice, models of AD, sodium butyrate exhibited a profound effect on improving learning and memory; expression of learning-associated genes was increased with butyrate treatment, probably by restoring histone acetylation profile [72, 73]. Although butyrate did not affect wild-type mice, it markedly improved contextual memory in the transgenic mice model, even at late stages of Alzheimer’s degeneration. SCFAs are produced in the fermentation process by intestinal bacteria; it is intriguing to see if the high-fiber diet has a positive effect on cognitive functions in humans. On several studies in healthy children fed a diet containing more fibers significantly better cognitive control was observed, compared to children who ate a regular, low-fiber diet [74]. Administration of probiotic bacteria lowered psychological stress in people [75] and reduced anxiety in chronic fatigue syndrome [76]. Undoubtedly, the restoration of probiotic flora, planned modulation of its composition, and dietary intervention to increase the function of probiotic bacteria in SCFAs production, remain promising interventions that, if carefully verified, can significantly improve the therapeutic perspectives of many brain diseases, including dementia and AD.

A wide variety of microbiome-resident bacteria and fungi generate significant quantities of LPS, amyloids, and various microbial exudates [57, 77, 78]. Amyloid is a generic term for any aggregated, insoluble, lipoprotein rich deposit exhibiting β-pleated sheet structures oriented to the fibrillar axis [77, 79]. Many bacterial species continuously produce and release extracellular protein fibers to create and maintain a biofilm, as such fibers offer protection from environmental stresses as well as mediate adherence to both biotic and abiotic surfaces [78, 80]. Microbial and cerebral amyloids are biologically similar regarding higher-order structure and pathogen-associated molecular pattern (PAMPs) composition and physic-chemical characteristics, although they do not share amino acid sequences with human Aβ1–42 [77]. However, they are recognized by the same TLR2/TLR1 receptor system as Aβ-42, and also strongly activate the production of pro-inflammatory cytokines, particularly IL-17 and IL-22. It should be emphasized that also in the state of health, man is exposed to extremely large amounts of LPS and amyloid proteins produced continuously by human microbiome [57]. Such exposure may be dangerous to health, especially in the course of aging, when the gastrointestinal mucosa, as well as the BBB, become significantly restructured and permeable [57, 77, 81]. Summarizing, total microbiome—derived secretory products constitute a large class of very powerful pro-inflammatory complement and innate immunity activators, that have enormous potential to induce pro-inflammatory cytokines, complement activation, and altered immunogenicity in the brain. Such pathogenic action enhances amyloid aggregation and significantly elevated inflammatory reactions. Both amyloid proteins and LPS are strong activators of the receptor for advanced glycation end-products (RAGE) and TLRs, and co-activation of these receptors amplify inflammatory signaling being an important driving force of sustained chronic inflammation in AD [82].

Aging is associated with marked changes in human microbiome, and age-related changes in gut microbiota composition involve a reduction of microbial biodiversity, with a higher abundance of Proteobacteria together with decreased number of Bifidobacteria species, and significantly lowered SCFAs production [83–85]. Age-related change of gut microbiome composition is most likely associated with chronic inflammatory reactions, which are common features in older people [83]. The urgent need to develop a therapy capable of inhibiting the development of neurodegeneration, including AD, aroused interest in ancient methods of transferring the intestinal microbiome from healthy people to the sick. For example, fecal microbiota transplantation is now increasingly used in people treating recurrent opportunistic infections of *Clostridium defficile* with very good clinical effects [86]. Also, studies in mice showed that fecal transplantation between animals was able to transfer behavioral traits between mouse strains [87]. Fecal transplantation alters brain chemistry and behavior in recipient ex-germ-free mice, raising the possibility of using this method of gut microbiome restoration for disorders of the CNS [83, 87]. It can be expected that transplantation of fecal microflora from healthy people to patients with AD can help restore intestinal microflora and reduce the negative impact of the dysbiotic microbiome on the gut and brain function.

The role of microbes in both aging and the onset and progression of the AD has been emerging in recent years. Studies on animals showed that modifications of gut microbiota induced by oral bacteriotherapy reflect changes in genes involved in inflammatory and neural plasticity processes with a positive impact on neural functions [88]. Improvement of cognitive function is supported by increased plasma concentration of gut hormones such as ghrelin, leptin, glucagon-like peptide-1 (GLP1), and gastric inhibitory polypeptide (GIP). This peptide hormone secreted in the gut plays a role in modulating nerve functions like learning and memory [89]. Mice treated with probiotic mixture showed higher plasma levels of such hormones, and this is important because ghrelin has been proven to counteract memory deficits and synaptic degeneration in AD animal models [90], and leptin has been demonstrated to act as neurotrophic factor and to exert neuroprotective effects against toxicity induced by Aβ oligomers in vitro [91]. It is confirmed that modification of microbiota produced anti-inflammatory effects in probiotic administered subjects. Treated AD mice possess higher levels of granulocyte colony-stimulating factor (G-CSF) that is a modulator of systemic immune responses by inhibiting pro-inflammatory cytokines and has been demonstrated to decrease Aβ deposition and to reverse cognitive impairment in an AD mice model [92]. New evidence utilizing Pan Cytokeratin immunostains of AD brain tissue demonstrates that these tissues contain parasites-like nematode larval and adult worms (and worm eggs), which were never found in normal brain tissues. Microbial proteins secreted by nematodes may influence neurodegeneration through the promotion/inhibition of amyloid formation by human proteins or by enhancing inflammatory responses to endogenous neuronal amyloids [93].

On the other hand, it is suggested that the intestinal nematodes and their secreted individual immune modulatory molecules may be considered as a defined therapeutic strategy. Successful infection by gastrointestinal nematodes results from their ability to survive within their hosts for long periods and causes chronic infection through regulation of the host immune responses, physiology, and behavior [94]. In long-lasting infection, nematodes produce and release factors which may actively modulate immune reactions and mimic host molecules or factors [94]. Intriguingly, recent studies suggest that the immunoregulatory capacity of worms may to some extent be directly or indirectly related to alterations in intestinal microbial communities, and the re-establishment of this balance by reintroducing gastrointestinal nematodes into patients is proposed as a novel potential immunotherapy [95]. Although no direct relationship has yet been demonstrated, the application of intestinal nematodes as a modulator of the microbiota–gut–brain axis may represent a promising strategy for the treatment of multiple sclerosis, [96], autism spectrum disorder [97], and other CNS disorders such as Alzheimer’s type neurodegeneration.

Intestinal nematodes reside in an extraordinary environment with commensal intestinal microbiota, where any disturbances result in the dysregulation of the immune system, thus leading to the development of chronic inflammatory disorders of the intestinal tract and even the CNS. Intestinal nematodes and host commensal flora, gut bacteria, sharing the same environmental niche evolved together [98]. It is quite possible that, as hosts, parasites benefit from the metabolic functions of the gut microbes. Given the pivotal roles that disturbances in the intestinal microbiota play in multiple autoimmune disorders, it is likely that the helminths can directly or indirectly contribute to reinstating the gut homeostasis by modulating the composition of the host gut microbiota to restore a “healthy” flora and provide more favorable conditions for its establishment in an adverse and aggressive inflammatory environment. Mutual adaptation of intestinal parasites and the intestinal microbiota is likely to also have consequences for other inflammatory diseases or for host physiology outside the immune system [99]. Similar alterations in the metabolic potential of intestinal bacterial communities have been observed in diverse parasitic and host species, suggesting that it represents an evolutionarily-conserved mechanism of host-microbe-helminth interactions [100, 101]. The changes in the gut microbiota under nematode infection may be a consequence of the secretion of antimicrobial components by the parasite that actively modifies the microbiota, or the disruption of the epithelial barrier by the parasite, which alters the intestinal environment and favors the establishment of selected commensals, or the stimulation of specific immune responses, e.g., expansion of regulatory T cells (*T*_regs)_ that actively contribute to a shift in gut microbiota [102]. It is plausible that helminths can control the microbiome directly. The worm expresses antifungal or antibacterial polypeptides when under attack by pathogens, as demonstrated by free-living nematodes such as *Caenorhabditis elegans* [103]. The last experimental study of Ramanan et al. [104] provides new insight into the speculations by showing that nematode infection with *Heligmosmoides polygyrus* can prevent *Bacteriodes vulgatus* outgrowth and protect mice against intestinal inflammation by restoring goblet cell numbers in Nod2−/− mice [104]. The helminth-mediated prevention of *B. vulgatus* results from the type 2 immune response that leads to the expansion of intestinal bacteria Clostridiales species [104]. Based on the study, we can speculate that the therapeutic properties of nematodes are partly associated with their ability to promote species richness and restore/maintain microbial homeostasis in the gastrointestinal tract.The multidimensional relationships between the host body, gut microbiota, and parasites result in the formation of a complex ecosystem where alterations in one of the these components determine a counter response in the others. Thus, the microbiota may impact on parasite burdens through observed alterations of intestinal physiology. Although a direct correlation needs to be confirmed, it is very plausible that the dysbiosis of gut microbiota influences nematode establishment, as higher establishment is only observed in cases of intestinal inflammatory disorder [105], but not in animals with peripheral inflammation occurring in response to encephalomyelitis [106]. The interactions between gastrointestinal helminths and commensal bacteria are likely to play a pivotal role in the establishment of host-parasite cross-talk, where commensal bacteria and helminths interact and provide signals that impact their survival in the host. For this reason, the successful manipulation of intestinal worms based on unique strategies of manipulating both the host and its commensal community could have remarkable therapeutic consequences given that gut microbiota dysbiosis affects immune activity in the CNS.

**Fig. 2:**
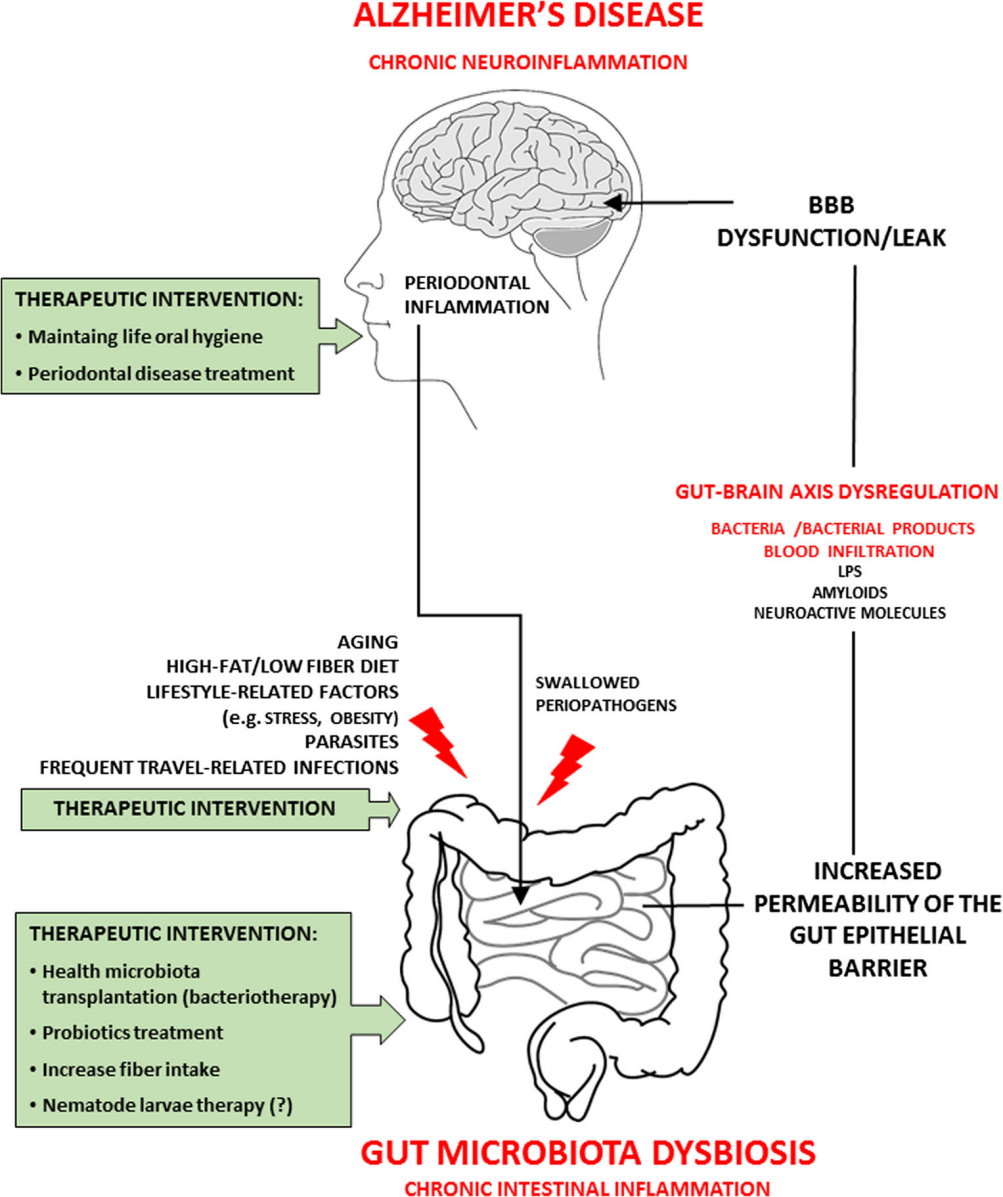
Gut microbiota dysbiosis and Alzheimer’s disease. Mechanism and potential therapeutic interventions. The gut-brain-axis—a pathway which gut microbiota can modulate host brain function and behavior. Aging, diverse lifestyle-related risk factors, as well as different infections can induce alterations of gut microbiota [dysbiosis] increasing risks of neurodegeneration disorders such as Alzheimer’s disease. Pathogenic bacteria and their products [LPS, amyloids, nueroactive molecules] may induce increased permeability of intestine epithelial barrier and blood-brain barrier dysfunction/leak that may induce/support chronic inflmmatory reactions in the brain. From the times of Hippocrates, it is believed that there is an association between mental health and intestinal flora imballance. Possibly, modulation of gut microbiota as well as therapeutic intervention against gut microbiota dysbiosis will become a new approach to treatment for AD

Currently, presented methods can be considered as a real prospect of slowing down the development of Alzheimer’s type neurodegeneration (Fig. 2).

## Conclusions

The lack of effective treatment for Alzheimer’s disease stems mainly from the incomplete understanding of AD causes. Currently, there are several hypotheses which try to explain the early molecular mechanisms of AD pathogenesis. The current pathophysiological approach is based on a number of common known mechanisms of neurodegeneration, including accumulation of abnormal proteins tau an ABeta, mitochondrial dysfunction, oxidative stress, impaired insulin signaling, calcium homeostasis dysregulation, imbalance of neurotransmitters, early synaptic disconnection, late apoptotic cell death, and especially neuroinflammation in CNS with dysfunction of microglia, the brain resident macrophages. AD pathophysiology problem have long term considered as the diseases only of CNS without important influence of the periphery.

Meanwhile, the rapidly growing number of scientific reports highlights the important role of peripheral infections and intestinal bacterial flora in the physiological function of the microbiome-intestine-brain axis. Microbiome controls the basic aspects of CNS, immunity, and behavior in health and disease. Changes in the amount and composition of the microbiome (dysbiosis) can be linked to disorders of the immune, endocrine, and nervous system, including mood changes, depression, increased susceptibility to stressors, and autistic behaviors. Abnormalities in the composition of the intestinal microbiome have also been found in many neurodegenerative diseases, including Alzheimer’s neurodegeneration. Dysbiotic intestinal microbiome, often accompanied by fungi and parasitic worms, jointly produces and releases onto the surface of the intestinal mucosa a multi-component mixture of secretion substances and microbial metabolic products containing a large group of compounds significantly increasing the innate immune function, elevating production of cytokines and inflammatory mediators. These compounds further increase the permeability of the intestinal mucosa and the permeability of the blood-brain barrier, significantly intensify inflammatory reactions, and induce amyloid aggregation. Decreased tightness of the intestinal mucosa and increased permeability of blood-brain barriers in the elderly, as well as dysbiosis of the intestinal microbiome, facilitate the entry of a large amount of bacterial amyloid and LPS into the circulatory system and CNS, where they activate innate resistance receptors: TLR and RAGE. Cooperation and cross-reactivation of RAGE and TLR receptors maintain a neuropathogenic inflammatory cycle in neurodegenerative diseases, including AD and other neuropathological disorders with the amyloidogenic component.

It is more likely that infections, infectious agents, and their toxic products may be a trigger factor for neurodegenerative processes, mainly through disruption of functioning of the immune system, which is associated with excessive synthesis and accumulation of Aβ, hyperphosphorylation of tau protein, and induction of chronic inflammation in the brain. Chronic peripheral infections and dysbiotic intestinal microflora may lead to many pathological changes in different tissues, also distant from the source of infection. Initially, the body is able to resist of this alterations; however, during aging, the regenerative capacity and ability to restrict infections decrease, leading to predominate of neurodegenerative processes and clinical manifestations of dementia. This confirms that pathological changes in the brain may start and follow many years before symptoms of the disease appear.

Early human studies suggested that altering microbiota with beneficial bacteria or probiotics can lead to changes in brain function including cognitive functions. Recently, identification of gastrointestinal microorganisms using metagenomics and metabolomics methods increased ability to examine more subtle interactions between host and microbiome. The gut provides the largest physical interface between the environment (including the microbiome) and self. This bidirectional information flow between the microbiota and the brain suggests that the brain development, function, mood, and cognition may be influenced by our gastrointestinal contents. We should underline that microbiota with major component as bacteria can induce autoimmune and widespread neuroinflammation disorders with aging such AD. Our experiences and review of literature allow us to pay attention to the other relatively less- until recently -operated field of possible pathology which serious and perhaps predominant influence on the development of pathophysiological changes in the brain of AD patients.

According to our opinion, in the near future, better understanding of the bidirectional communication between the brain and microbiota, host-microbe-helminth interactions, and determining the role of specific probiotic bacteria will allow the development of functional diets and the use of intestinal microbiome to improve the results of pharmacological therapy of many diseases. It is expected that these pathways will be especially harnessed to provide novel method to enhance health and treat AD. There is no doubt that in patients with AD, attempts to restore the intestinal microbiome to a composition reminiscent of that found in healthy adult humans will significantly slow down the progression of neurodegeneration by lowering the level of inflammatory reactions and/or amyloidogenesis.

In conclusion, the new strategies seem to focus on examining the potential neuroprotective activity of disease-modifying drugs in the presymptomatic stages of AD, with the help of biomarkers that predict disease progression before development of overt dementia.
